# Self-Reported Prevalence of Gluten-Related Disorders and Adherence to Gluten-Free Diet in Colombian Adult Population

**DOI:** 10.1155/2016/4704309

**Published:** 2016-08-28

**Authors:** Francisco Cabrera-Chávez, Diana María Granda-Restrepo, Jesús Gilberto Arámburo-Gálvez, Alejandro Franco-Aguilar, Dalia Magaña-Ordorica, Marcela de Jesús Vergara-Jiménez, Noé Ontiveros

**Affiliations:** ^1^School of Nutrition Sciences, Universidad Autónoma de Sinaloa, Av. Cedros y Calle Sauces S/N, Fracc. Los Fresnos, 80019 Culiacán, SIN, Mexico; ^2^Departamento de Alimentos, Facultad de Ciencias Farmacéuticas y Alimentarias, Universidad de Antioquia, Calle 67 No. 53-108, Ciudadela Universitaria, Medellín, Colombia; ^3^Regional Program for Ph.D. in Biotechnology, FCQB, Universidad Autónoma de Sinaloa, Josefa Ortiz de Domínguez, S/N, Ciudad Universitaria, 80013 Culiacán, SIN, Mexico

## Abstract

*Background*. Celiac disease seems to be rare in Colombians, but there are currently no data about the prevalence rates of symptomatic adverse reactions to gluten or adherence to gluten-free diet (GFD) in this population.* Aim*. to evaluate the self-reported prevalence rates of adverse reactions to gluten, adherence to GFD, and gluten-related disorders at population level in Colombia.* Methods*. A self-administered questionnaire-based cross-sectional study was conducted in a population from Northwest Colombia.* Results*. The estimated prevalence rates were (95% CI) 7.9% (6.5–9.6) and 5.3% (4.1–6.7) for adverse and recurrent adverse reactions to wheat/gluten, respectively, adherence to GFD 5.9% (4.7–7.4), wheat allergy 0.74% (0.3–1.4), and nonceliac gluten sensitivity 4.5% (3.5–5.8). There were no self-reported cases of celiac disease. Prevalence of self-reported physician-diagnosis of gluten-related disorders was 0.41% (0.17–0.96). Most respondents reported adherence to GFD without a physician-diagnosis of gluten-related disorders (97.2%). The proportion of gluten avoiders was 17.2% (15.2–19.5). Most of them did not report recurrent adverse reactions to wheat/gluten (87.0%). *Conclusions*. Nonceliac gluten sensitivity is rarely formally diagnosed in Colombia, but this population has the highest prevalence rate of adherence to GFD reported to date. Consequently, most respondents were avoiding wheat- and/or gluten-based products for reasons other than health-related symptoms.

## 1. Introduction

Celiac disease is an autoimmune inflammatory condition and its clinical presentation of celiac disease can fluctuate from asymptomatic to severe malabsorption. Other gluten-related disorders, such as wheat allergy and nonceliac gluten sensitivity (NCGS), are symptomatic conditions without an autoimmune background. Particularly, due to symptoms largely overlapping, symptomatic celiac disease and NCGS cases cannot be distinguished clinically. Furthermore, based on laboratory tests, discerning between celiac disease and NCGS in adults could be difficult as celiac diagnostic markers, such as serological tests and histology, do not appear as clear in adults as in children. In either case, symptomatic relief is reached after gluten withdrawal. Consequently, although wheat allergy cases could be better suited for a wheat-free diet, symptomatic gluten-related disorders are linked to symptomatic adverse reactions to oral gluten and a gluten-free diet (GFD).

The GFD health benefits are limited to those who have a diagnosed gluten-related disorder. However, especially in adults, adhering to a GFD without proper dietary advice could negatively have an impact on both dietary intake [1] and serum levels of micronutrients such as iron and vitamin B [2–4]. Furthermore, since celiac disease serology and histology should be performed while the patients are still on a gluten containing diet and this condition should be excluded in the diagnostic work-up of NCGS, adhering to a GFD without a proper diagnosis of these disorders could complicate their diagnostic work-up [5, 6]. Therefore, dietary restriction of wheat and/or gluten should only be instructed in those with a formally diagnosed gluten-related disorder.

Celiac disease prevalence in Latin America ranges between 0.46% and 0.64% [7], but the prevalence of gluten-related disorders prevalence is still unknown in this region of the world. The first step to objectively estimate the prevalence rates of wheat allergy or NCGS at the population level is the eligibility of individuals that develop symptomatic adverse reactions to wheat and/or gluten. Accordingly, we have developed an instrument to estimate these prevalence rates in the Spanish-speaking Latin population [8]. This instrument enabled us to highlight that gluten-related disorders are subdiagnosed in the Mexican population, but more people than those that truly benefit from a GFD may be avoiding wheat- and/or gluten-based products from their diets [8]. This could point out that the Latin population with no a formally diagnosed gluten-related disorder has a growing interest in adhering to a GFD, similar to other countries from North America, Oceania, and Europe [9]. However, neither adherence to a GFD nor adverse reactions to oral wheat and/or gluten prevalence rates have been estimated at population level in Central and South America and islands of the Caribbean.

Particularly, celiac disease seems to be rare in Colombians [7] and this could be related to a low prevalence rate of adverse reactions to oral wheat and/or gluten. On the contrary, due to the potential growing enthusiasm for adhering to a GFD, the prevalence of adherence to this diet in Colombia could be as high as in other countries. Thus, our aim was to estimate the prevalence rates of self-reported adverse reactions to oral wheat and/or gluten, adherence to a GFD, and gluten-related disorders in the Colombian adult population.

## 2. Materials and Methods

### 2.1. Population Survey

We conducted a population-based cross-sectional survey in Antioquia, Medellin, Colombia. All data were collected during the period from February to March 2016. All respondents were approached in urban parks of Antioquia and outside shopping malls and supermarkets located in the downtown area of Medellin. Inclusion criteria were as follows: subjects aged 18 years or older and subjects being able to read and answer the questionnaire by themselves. Exclusion criteria included subjects less than 18 years or subjects that were not able to complete the questionnaire by themselves. Trained nutritional science students gave assistance on specific terms when it was requested.

### 2.2. Questionnaire

A previously validated Spanish version of a self-administered questionnaire was utilized in this study [8]. Respondents first answered questions related to basic demographics and clinical information. This part also includes a key question about adverse reactions to wheat and/or oral gluten (Do you have some discomfort or adverse reaction when consuming wheat products?). The first section included 15 questions and was designed for those who reported adverse reactions to wheat and/or oral gluten. In this section, the respondents answered questions about the diagnostic work-up of gluten-related disorders (Have you ever been diagnosed with a disease related to the consumption of wheat or gluten?) and standardized questions about symptoms suggestive of IgE-mediated food allergy and time of appearance of the symptoms after food ingestion. The second section includes 14 questions and was designed for those who reported adverse reactions to other foods different from wheat/gluten. The first and second sections of the instrument collected information about gastrointestinal and extraintestinal symptoms and symptoms frequency, among others. All participants answered the question: do you keep a diet free of wheat and/or gluten? And a question related to gluten avoidance was answered (Do you avoid, as much as possible, wheat- or gluten-based products from your diet?).

### 2.3. Definitions

An adverse food reaction was defined as any symptomatic adverse food reaction to a specific food potentially mediated or not by immune mechanisms [8]. Recurrent adverse reactions were considered when the respondents reported that the food-induced symptoms occurred always or most of the times. Self-reported physician-diagnosed celiac disease was considered when the respondents reported that they were diagnosed by a physician and were also following a GFD [10]. These criteria also applied for a reported diagnosis of wheat allergy. In addition, self-reported wheat allergy was considered when the respondents reported recurrent adverse reactions “convincing” of food allergy. This includes skin with hives and angioedema, trouble breathing, wheezing or throat tightness, and vomiting and diarrhea, and the symptoms occurred within 2 h after ingestion of food [11–14]. Self-reported physician-diagnosed NCGS was considered when the respondents stated that a physician diagnosed them. Self-reported NCGS was defined as those cases that met the following: (a) respondents without a known diagnosis of celiac disease or wheat allergy, (b) respondents that did not meet criteria for self-reported wheat allergy, and (c) respondents that met criteria for recurrent adverse reactions to wheat and/or gluten.

### 2.4. Statistical and Ethical Issues

Statistical analysis was carried out using PASW statistics version 18.0 (SPSS Inc., Chicago, Illinois, USA). Categorical variables were summarized by descriptive statistics, including total numbers, percentages, odds ratio, and 95% confidence interval. Associations were analyzed by two-tailed Fisher's exact test. Continuous variables were summarized by mean and range with differences between two groups calculated using the Student *t*-test. *p* value < 0.05 was considered statistically significant. Prevalence rates were calculated using OpenEpi software version 3.03a (http://www.openepi.com/, updated 2015/05/04). Rates were reported as rate (95% confidence intervals) per 100 inhabitants. All respondents received sufficient information and gave informed consent in writing to participate in the study. The study protocol was approved by the Ethics Review Board of the University of Antioquia (Universidad de Antioquia [ethic approval number: Acta 001 de 2016]).

## 3. Results

### 3.1. Study Participants and Demographic Characteristics

A total of 1314 individuals were approached. Of these, 1207 agreed to participate in the survey (response rate, 92%). The proportion of females was higher than male (27.3%) (*p* > 0.05). The demographics and clinical characteristics of the studied population are given in Table 1. The most common self-reported physician-diagnosed disorders were lactose intolerance (9.4%), irritable bowel syndrome (IBS) (8.9%), and nonfood allergies (8.0%), followed by food intolerance (other than lactose intolerance) (4.6%) and colitis (3.9%). IBS, eating behavior disorders, and colitis were significantly more common in women than in men (*p* < 0.05).

### 3.2. Characteristics of Subjects with Self-Reported Recurrent Adverse Reactions

The characteristics of respondents with recurrent adverse reactions to wheat and/or gluten and those with recurrent adverse reactions to foods other than gluten are shown in Table 2. Although most of the diseases assessed (6 out of 9) were more common in those who self-reported recurrent adverse reactions to gluten than the opposing group, there was no difference in disease or demographics between the groups (*p* > 0.05). Similarly, the absence of disease was more common in the group of recurrent adverse reactions to other foods different from gluten than in the group of recurrent adverse reactions to gluten (49.4% vs 37.5%) and the presence of two or more diseases was more common in this last group than in the opposing one (20.3% vs 14.2%), but this was not statistically significant in both cases (*p* > 0.05). However, the risk analyses showed odds ratios of 3.4 (95% CI; 0.74–15.6) and 2.2 (95% CI; 0.54–9.2) for psychiatric disease and eating behavior disorder, respectively (Table 2). Further analysis between self-reported NCGS and those who did not meet criteria for self-reported NCGS showed significant differences for IBS (*p* < 0.001), food intolerance (*p* < 0.007), and eating behavior disorders (*p* < 0.016).

### 3.3. Prevalence Rates of Adverse Food Reactions, Gluten-Related Disorders, and Adherence to GFD

Of the total population surveyed, there were no self-reported physician-diagnosed celiac disease cases. Other prevalence estimations are shown in Table 3. Overall, 18.7% (64 out of 342) of the recurrent adverse food reactions cases were attributed to the consumption of wheat- and/or gluten-based products, 16.0% (55 out of 342) met criteria for NCGS, and 2.6% (9 out of 342) for wheat allergy. Except for adverse food reactions and wheat allergy prevalence rates, all prevalence estimations were significantly higher in women than in men (*p* < 0.05).

**Figure 1:**
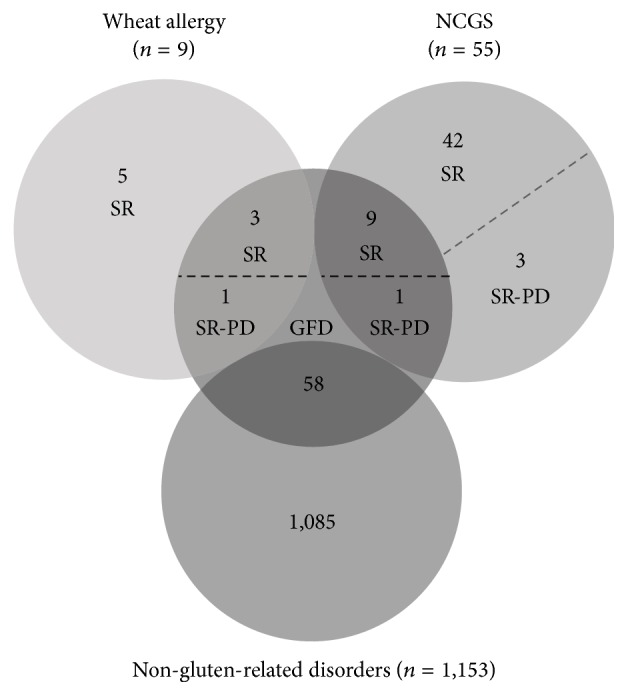
Characteristics of respondents adhering to a GFD. NCGS: nonceliac gluten sensitivity; SR: self-reported wheat allergy or NCGS; SR-PD; self-reported physician-diagnosed wheat allergy or NCGS. General practitioners diagnosed the five SR-PD cases.

Previous studies estimated the prevalence rates of NCGS based on GFD adherence [15]. In this study, 55 respondents met criteria for NCGS and 9 for wheat allergy, but only 10 and 4 cases were on a GFD, respectively. Thus, the general prevalence rates of NCGS or wheat allergy currently adhering to a GFD are 0.82% (95% CI; 0.45–1.51) and 0.33% (95% CI; 0.13–0.85), respectively. Among those that met criteria for gluten-related disorders (*n* = 64), only 14 (21.8%) were on a GFD and 5 met criteria for self-reported physician-diagnosed gluten-related disorders (0.41%; 95% CI; 0.17–0.96) (Figure 1).

**Figure 2:**
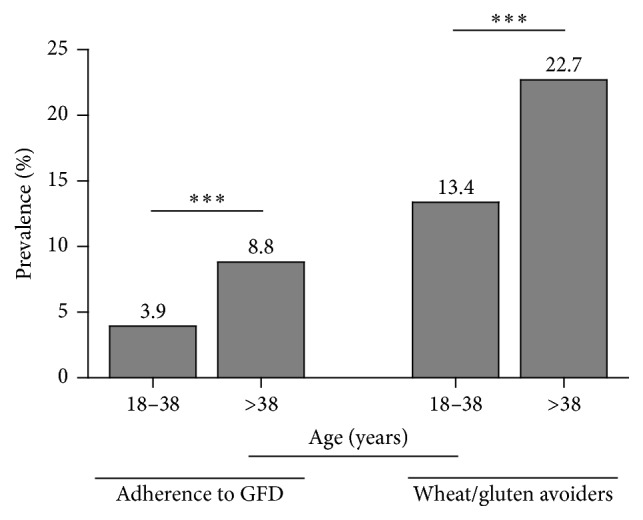
Prevalence of adherence to a GFD and wheat/gluten avoidance stratified by age (years). ^*∗∗∗*^ *p* < 0.001.

Next, we analyzed the self-reported GFD and gluten avoidance cases. General prevalence of wheat and/or gluten avoidance was 17.2% (*n* = 208; 95% CI; 15.2–19.5). The prevalence rates of adhering to GFD or gluten avoidance were significantly higher among respondents <38 years than those >39 years (*p* < 0.001) (Figure 2). Of the 208 respondents who were avoiding gluten from their diets, 49 (23.5%) and 159 (76.5%) reported adverse reactions to wheat and/or gluten or to other foods, respectively. Notably, only 27 (12.9%) reported recurrent adverse reactions to wheat and/or gluten, while 97 (76.4%) reported no adverse reactions either to wheat and/or gluten or to other foods. Regarding the GFD cases, only 13 (18.0%) out of 72 reported recurrent adverse reactions to wheat and/or gluten. Other 9 cases (12.5%) on a GFD reported occasional adverse reactions to wheat and/or gluten. Therefore, most of the gluten avoiders reported no adverse reactions either to foods (*n* = 25; 34.7%) or to other foods different from wheat/gluten (*n* = 25; 34.7%). Overall, these data show that most Colombians on a GFD or trying to avoid wheat- and/or gluten-based products from their diets are doing it for reasons other than health-related symptoms.

### 3.4. Self-Reported Symptoms in NCGS Cases

**Figure 3:**
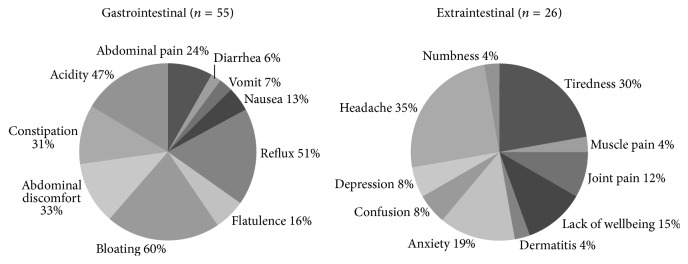
Self-reported NCGS symptoms.

The symptoms reported by those who met the criteria for NCGS are shown in Figure 3. All NCGS cases identified (55) reported gastrointestinal symptoms and 26 (46.4%) out of 55 reported extraintestinal symptoms. The mean age when the symptoms related to gluten intake appeared was 33.9 (range: 14–78) and 29.5 years (range: 15–57) for gastrointestinal and extraintestinal symptoms, respectively (*p* > 0.05). The most common gastrointestinal symptoms were bloating (60.0%), reflux (51.0%), and acidity (47.0%), followed by abdominal discomfort (33.0%) and constipation (31.0%) (Figure 3). In those who reported extraintestinal symptoms, the most common manifestations were headache (35.0%), tiredness (30.0%), and anxiety (19.0%), followed by lack of wellbeing (15%), and joint pain (12.0%) (Figure 3). Symptom comparisons between NCGS cases and those who reported recurrent adverse reactions to other foods showed statistically significant differences for abdominal pain (24.0% versus 45.0%), flatulence (16% versus 40%), diarrhea (6% versus 23%), and constipation (31% versus 18%) (*p* < 0.05).

## 4. Discussion

A population-based survey was conducted to evaluate the prevalence of self-reported gluten-related disorders and adherence to a GFD in the Colombian adult population. As some conditions such as IBS, dairy intolerance, and autoimmune or eating behavior disorders have been associated with gluten sensitivity in adult Europeans [10, 16], we collected clinical information about the studied population. Similar to previous research [8], in this study, there were no significant associations between self-reported recurrent adverse reactions to gluten or NCGS cases and those who reported recurrent adverse reactions to other foods different from gluten. However, comparisons between self-reported NCGS and those who did not meet criteria for self-reported NCGS showed significant differences for IBS, food intolerance, and eating behavior disorders, similar to other survey studies carried out in Europe, which screened for symptoms consistent with IBS in accordance with Rome III criteria [10]. Overall, our data support the notion that some conditions such as IBS and food intolerance are more common in self-reported NCGS than in the general population, but these conditions seem to be equally common in self-reported NCGS and in those who reported recurrent adverse reactions to foods other than wheat and/or gluten.

Prevalence estimations of adverse reactions to wheat and/or gluten, either recurrent (5.3%) or not (7.9%), were lower than those reported in the Mexican population (7.8% and 11.9%, resp.) [8]. Despite these differences, NCGS prevalence is higher in Colombians than in Mexicans (4.5% vs 3.3%), considering even the self-reported NCGS cases currently consuming a GFD (0.82% vs 0.16%) [8]. Certainly, NCGS prevalence largely varies among studies and our results are in line with previous NCGS prevalence estimations, which typically fluctuate between 0.55% and 13.0% [10, 15–17].

Celiac disease prevalence in Latin America fluctuates between 0.46% and 0.64%, but this condition is particularly rare in Colombians [7]. In the present study, there were no self-reported cases of celiac disease in the Colombian adult population surveyed. As celiac disease has a strong genetic background and gluten exposure is essential to trigger the condition, these factors should be linked to the low celiac disease prevalence rate in Colombians. The European ancestry alleles HLA-DQA1*∗*05:01 and –DQB1*∗*02:01 encoding the haplotype HLA-DQ2.5 show the highest genetic risk for celiac disease [5, 18]. European ancestry alleles could be more common in Colombian mestizo from Medellin than mestizo from another regions of Colombia or Latin America [19], but precise information describing the prevalence of celiac disease risk alleles in the study population is scarce. Regarding wheat consumption, more than 90% of Colombians consume wheat-based products like pasta or bread daily [20], but wheat consumption in Colombia is lower than in Mexico and most South American countries [21] and European ones [18]. Therefore, genetic and environmental factors as well as a potential subdiagnosis of celiac disease in Latin population [8] are facts that could explain the scarcity of celiac disease cases in Colombia.

Regarding wheat allergy, the estimated prevalence of self-reported wheat allergy in Colombians was quite similar to that reported in the Mexican population (0.74% versus 0.72%, resp.) [8]. Similar prevalence rates of self-reported wheat allergy have been described in other countries outside Latin America [22–24]. Overall, considering that celiac disease is a rare condition in Colombians [7] and typical wheat allergy symptoms in adults are easily recognized [5], our results highlight that NCGS, which is a form of gluten intolerance involving the exclusion of celiac disease and wheat allergy for its diagnosis [5, 6, 17], is a condition rarely formally diagnosed in Colombia.

The self-reported prevalence of adherence to a GFD was 5.9%. This is the highest prevalence ever reported at population level, either in children [25–27] or in adults [8, 10, 25, 26, 28]. Most notably, it was higher than the prevalence rate of symptomatic recurrent adverse reactions to oral wheat and/or gluten in the Colombian population surveyed (5.3%). However, adhering to a strict GFD is challenging even for formally diagnosed celiac disease patients [29] and it is possible that some respondents that reported to be following a GFD were accidentally exposed to oral gluten.

The proportion of wheat and/or gluten avoiders was 17.2%, which is higher than the prevalence rate of adverse reactions to wheat and/or gluten (7.9%) reported in this study (7.9%) and also than the proportion of gluten avoiders reported in Australian population (10.6%) [28]. Reasons for adopting a GFD include perceived benefits on health, personal taste or preference, weight loss, and treating disease and/or minimizing future risk of disease [28, 30]. The high proportions of individuals adhering to a GFD and gluten avoiders in Colombia indicate that most individuals were currently avoiding wheat- and/or gluten-based products for reasons other than health-related symptoms. However, in the absence of gluten-related disorders, adhering to a GFD is unlikely to present health benefits [31].

Previously [8] and in the present study, we evaluated the self-reported symptoms triggered by wheat/gluten intake. The main findings included the following: (a) the most common self-reported gluten-associated symptoms were comparable to previous studies, despite the different approaches or instruments that were utilized [10, 16], (b) the main self-reported symptoms triggered by wheat/gluten intake are not truly representative as they were also frequent or unusual in those who reported recurrent adverse reactions to other foods different from wheat/gluten, and (c), in a clinical setting, symptoms evaluation* per se* is not enough to attribute the manifestations to gluten intake, and objective diagnostic criteria such as celiac serology/histology or oral food challenges are required to make definitive conclusions. Since the main gluten-related and non-gluten-related symptoms reported by Colombians, either intestinal or extraintestinal, were reproducible from previous research [8], the present study properly confirms those findings.

The strengths of our study include its population-based design, the high response rate (92%) minimizing possible nonresponse bias, and the criteria used to estimate the prevalence rate of wheat allergy. It has been shown that most subjects fulfilling these criteria (93%) had IgE antibody to the implicated food [13]. However, it should be acknowledged that our study has some limitations. First, the use of self-reporting to estimate prevalence rates has been found to overestimate the real prevalence rates [32], and our data were not confirmed by more objective diagnostic studies such as skin prick tests, specific IgE antibodies, celiac serology and/or histology, or oral food challenges. Secondly, our study had a limitation in assessing the motivations for consuming a GFD and in determining who instructs the GFD. However, it provides useful information regarding the self-reported prevalence rates of symptomatic recurrent adverse reactions to gluten, adherence to a GFD, and gluten-related disorders in Latin population. Furthermore, it serves as groundwork for further studies based on objective diagnostic criteria.

## 5. Conclusion

The present study is the first to report the self-reported prevalence rates of symptomatic adverse reactions to gluten, adherence to a GFD, and gluten-related disorders at the population level in South American countries. We observed a low prevalence of physician-diagnosis of gluten-related disorders in the Colombian population studied, but this population also reported the highest prevalence of adherence to a GFD compared to previously published data. This highlights a growing enthusiasm for adhering to a GFD in the Colombian population, and that most are doing it without a formally diagnosed gluten-related disorder and probably without medical/dietary advice.

## Figures and Tables

**Table 1:** Demographics and clinical characteristics of the studied population.

Variable^*∗*^	—	*n*
Mean age in years (range)	37.6 (18–89)	—
Gender (female/male)	56%/44%	676/531
Lactose intolerance	9.4%	114
IBS^*∗∗*^	8.9%	108
Nonfood allergy	8.0%	97
Food intolerance	4.6%	56
Colitis	3.9%	48
Diabetes mellitus	2.5%	31
Psychiatric disease	1.9%	23
Eating disorders	1.0%	12
Food allergy	0.9%	11
Gastrointestinal cancer	0.4%	5

^*∗*^Self-reported physician-diagnosed diseases were considered for analysis.

^*∗∗*^Irritable bowel syndrome.

**Table 2:** Comparison between self-reported recurrent adverse reactions to wheat/gluten and recurrent adverse reactions to other foods.

Variable^*∗*^	Recurrent adverse reactions				Odds ratio (95% CI)
	Wheat/gluten (*N* = 64)		Other foods (*N* = 281)		
	—	*n*	—	*n*	
Mean age in years (range)^*∗∗*^	41.4 (18–78)	—	37.4 (18–88)	—	—
Gender (female/male) (%)	71.8/28.2	46/18	62.3/37.7	175/106	1.5 (0.853–2.8)
IBS^¶^ (%)	23.4	15	15.6	44	1.5 (0.76–2.9)
Food intolerance (%)	14.6	10	7.8	22	1.9 (0.84–4.41)
Allergy (%)	9.3	6	11.0	31	0.68 (0.25–1.8)
Psychiatric disease (%)	3.1	2	1.4	4	3.4 (0.74–15.6)
Gastrointestinal cancer (%)	1.5	1	1.0	3	1.4 (0.15–14.3)
Eating disorders (%)	4.6	3	2.1	6	2.2 (0.54–9.2)
Diabetes mellitus (%)	3.1	2	2.8	8	1.1 (0.22–5.3)
Colitis (%)	3.1	2	8.9	25	0.33 (0.07–1.4)
Lactose intolerance (%)	14.0	9	17.8	50	0.75 (0.35–1.6)

^*∗*^Self-reported physician-diagnosed diseases were considered for analysis.

^*∗∗*^Age comparison by Student's *t*-test (*p* > 0.05).

^¶^Irritable bowel syndrome.

**Table 3:** Self-reported prevalence rates estimations.

Assessment	(+) cases^*∗*^	Prevalence by gender (95% CI)	*p* value	General prevalence (95% CI)
Adverse food reactions	Total = 452 M^*∗∗*^ = 183 F^*∗∗*^ = 269	M 34.46 (30.5–38.6) F 39.8 (36.1–43.5)	0.063	37.4 (34.7–40.2)
Adverse reactions to wheat/gluten	Total = 96 M = 29 F = 67	M 5.4 (3.8–7.7) F 9.9 (7.8–12.4)	0.005	7.9 (6.5–9.6)
Recurrent adverse food reactions	Total = 342 M = 124 F = 218	M 23.35 (19.9–27.1) F 32.2 (28.8–35.8)	0.001	28.3 (25.8–30.9)
(i) Recurrent adverse reactions to wheat/gluten	Total = 64 M = 18 F = 46	M 3.4 (2.1–5.3) F 6.8 (4.1–8.9)	0.009	5.3 (4.1–6.7)
(ii) NCGS	Total = 55 M = 16 F = 39	M 3.0 (1.8–4.8) F 5.7 (4.2–7.7)	0.026	4.5 (3.5–5.8)
(iii) Wheat allergy	Total = 9 M = 2 F = 7	M 0.37 (0.1–1.3) F 1.0 (0.5–2.1)	0.313	0.74 (0.3–1.4)
Adherence to GFD	Total = 72 M = 22 F = 50	M 4.1 (2.7–6.2) F 7.4 (5.6–9.6)	0.020	5.9 (4.7–7.4)

^*∗*^Positive cases for the assessment.

^*∗∗*^F: female; M: male.
